# Radiological evaluation of pseudofracture after the administration of asfotase alfa in an adult with benign prenatal hypophosphatasia: A case report

**DOI:** 10.1016/j.bonr.2021.101163

**Published:** 2021-12-29

**Authors:** Hajime Kato, Naoko Hidaka, Minae Koga, Yuka Kinoshita, Noriko Makita, Masaomi Nangaku, Nobuaki Ito

**Affiliations:** Division of Nephrology and Endocrinology, The University of Tokyo Hospital, Japan

**Keywords:** Hypophosphatasia, Asfotase alfa, Pseudofracture, Alkaline phosphatase, Bone scintigraphy

## Abstract

Hypophosphatasia (HPP) is a congenital disorder with decreased activity of tissue-nonspecific alkaline phosphatase. Asfotase alfa is the only treatment approved for HPP and improves the impairment of bone mineralization. Although several previous studies have reported the efficacy of asfotase alfa to treat fractures and pseudofractures in patients with HPP, there are only a few reports with a detailed description of the healing process. In this case report, we present an 18-year-old female patient with benign prenatal HPP who received asfotase alfa to treat her pseudofracture. At the age of 17, a pseudofracture developed in her left tibia after repetitive gymnastic exercise for months. Following observation over a year, she was referred to our department. X-ray images indicated a narrow radiolucent band in the mid-diaphysis of her left tibia, and bone scintigraphy showed nuclide accumulation in the same region. Replacement therapy with asfotase alfa was started, resulting in pain relief in two months, and the disappearance of nuclide accumulation on bone scintigraphy and union of the pseudofracture on X-ray after two years. This is the first case report describing the detailed pseudofracture healing process in a patient with benign prenatal HPP initiating asfotase alfa.

## Highlights

• Previous data regarding the healing process of fractures and pseudofractures in HPP with asfotase alfa are limited.
• Clinical course of the healing process of pseudofractures in the tibia in benign prenatal HPP was radiologically followed.
• The nuclide accumulation in bone scintigraphy disappeared one year after the initiation of asfotase alfa.
• The fracture line visible by X-ray disappeared two years after the initiation of asfotase alfa.

## 1 Introduction

Hypophosphatasia (HPP) is an inborn error of metabolism and is caused by loss-of-function mutations in the *ALPL* gene, which encodes the tissue nonspecific isoenzyme of alkaline phosphatase (TNSALP) (Whyte, 2017). Inorganic pyrophosphate (PP_i_) is one of the substrates of TNSALP, and the accumulation of PP_i_ caused by the reduced enzymatic activity of TNSALP inhibits mineralization of the bone or leads to deposition of the pyrophosphate crystals in the joints (Fleisch et al., 1966; Guañabens et al., 2014; Russell et al., 1971; Russell, 1965). HPP presents a broad spectrum of clinical phenotypes, categorized as follows based on the onset and severity of the presentation: odonto-, adult, mild childhood, severe childhood, infantile, perinatal, and benign prenatal HPP (Whyte, 2016). While benign prenatal HPP shows severe skeletal dysplasia in utero similar to perinatal HPP, spontaneous improvement in mineralization and of the skeletal abnormalities occurs after birth, different from the ongoing skeletal dysplasia seen in perinatal HPP (Wenkert et al., 2011). In Japan, the carrier frequency of the most common frameshift mutation, c.1559delT in the *ALPL* gene, is estimated to be 1/480, and a homozygous c.1559delT mutation results in almost null enzymatic activity (Michigami et al., 2005). A missense mutation, c.979 T > C (F310L), with approximately 70% activity of wild-type TNSALP, was also a common mutation among Japanese individuals (Michigami et al., 2005; Watanabe et al., 2011). While patients with compound heterozygous c.1559delT and c.979 T > C (F310L) mutations in the *ALPL* gene often present benign prenatal HPP, patients with a homozygous c.1559delT *ALPL* mutation usually develop lethal perinatal HPP (Michigami et al., 2005; Watanabe et al., 2011).

Asfotase alfa (Strensiq®, Alexion Pharmaceuticals Inc., Boston, MA, USA), which improves the impairment of bone mineralization (Rolvien et al., 2019), is currently the only approved treatment for HPP. Since 2015, in Japan, the use of asfotase alfa has been approved for treating any type of HPP, including adult-onset HPP, while the use of this drug is restricted to patients with perinatal-, infantile- and juvenile-onset HPP in Europe and the United States. To date, many studies have reported the efficacy of asfotase alfa among infants, young children, adolescents, and adults with HPP (Kishnani et al., 2019; Whyte et al., 2016, Whyte et al., 2019). The main treatment goal for patients with perinatal and infantile HPP is the improvement of their survival rate via improvements of their respiratory status, skeletal deformity and seizure control. In addition, bone and joint pain, reduced mobility due to this pain and muscle weakness and the prevention of pseudofracture and fracture would also be treatment targets of asfotase alfa among perinatal and infantile HPP patients who survived their infancy and childhood, prenatal benign HPP patients, and adult-onset HPP patients in Japan (Kishnani et al., 2017). The indication of asfotase alfa in adult patients with HPP depends on their clinical presentations, and patients with fractures or pseudofractures have received ALP replacement to improve or prevent fractures/pseudofractures (Kishnani et al., 2017). Recently, Stürznickel et al. revealed the recalcification process after the initiation of asfotase alfa in three adults with pediatric-onset HPP with prolonged bone healing after arthrodesis, tibial stress fracture, and osteotomy using cone-beam computed tomography (Stürznickel et al., 2021). However, there are still few studies describing the detailed radiological changes during the healing process of fractures and pseudofractures in HPP patients after the initiation of asfotase alfa. We hereby present an adult patient with benign prenatal HPP with a pseudofracture in the left tibia, describing its healing process after the initiation of asfotase alfa until its complete union on X-ray and bone scintigraphy findings.

## 2 Case report

**Fig. 1:**
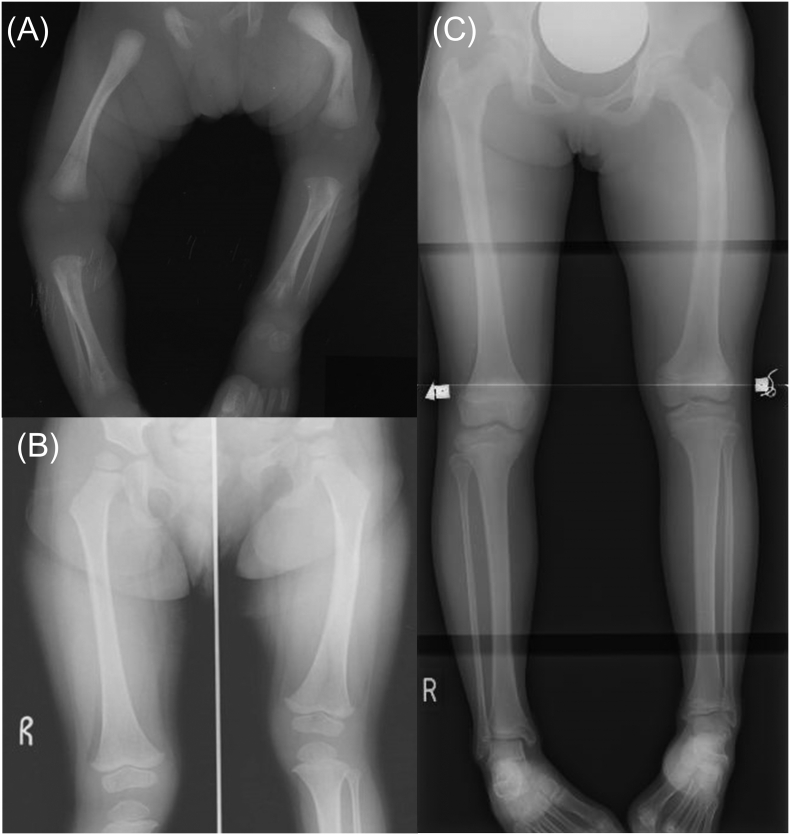
X-rays of the lower limb at birth (A), at two years of age (B), and at ten years of age (C). (A) Shortening and curvature of the left leg was observed at birth. (B, C) Discrepancy in leg length appeared at two years of age (B) and remained at ten years of age (C).

An 18-year-old Japanese female patient with HPP presented to our hospital with prolonged intensive pain in the left tibia for more than a year. Left femur shortening was detected by ultrasound and X-ray in her late fetal life (Fig. 1A), and low alkaline phosphatase activity (11 U/L, reference interval: 38–113) was recorded at birth. She was diagnosed with benign prenatal HPP at six months, with a genetic test revealing compound heterozygous mutations in the *ALPL* gene (c.1559delT and c.979 T > C). Because her leg length discrepancy remained at the age of 10 (Fig. 1B and C), percutaneous epiphysiodesis in the right femoral growth plate was performed. She suffered a fracture in the left upper limb during physical education class in elementary school at 12 and a pseudofracture in the left tibia after repetitive gymnastic exercise for months at 17. After pseudofracture developed, the patient stopped gymnastic exercise, but she did not use any equipment to reduce weight-bearing, such as a wheelchair or crutches. Because a year-long observation failed to achieve the union of the pseudofracture, she was referred to our department for the initiation of asfotase alfa. Her height was 1.54 m, which was −0.78 standard deviation score for the average height among age-matched Japanese individuals. Laboratory data indicated low ALP activity concomitant with high serum phosphate and high urinary phosphoethanolamine (PEA), which was compatible with a mild phenotype of HPP (Table 1). The level of bone alkaline phosphatase was analyzed by a chemiluminescent enzyme immunoassay (Beckman-Coulter, Brea, CA, USA). Osteocalcin was measured by an electrochemiluminescence immunoassay (Roche, Basel, Switzerland). Tartrate-resistant acid phosphatase 5b was assayed by an enzyme immunoassay (NAT Corporation, Ibaraki, Japan).

**Table 1:** Height, weight, biochemical profiles and physical tests of the patient before and after the initiation of asfotase alfa.

	Reference interval	Before asfotase alfa	One year with asfotase alfa	Four years with asfotase alfa
Height (m/SDS)		1.54 (−0.78)	–	–
Weight (kg/SD)		47 (−0.3)	–	–
Laboratory data				
Serum calcium (mmol/L)	2.1–2.5	2.3	2.3	2.3
Serum phosphate (mmol/L)	0.87–1.49	1.68	1.45(↓)	1.29(↓)
Alkaline phosphatase (U/L)	38–113	26	8495(↑)	1786(↑)
BAP (μg/L)	2.9–14.5	2.6	2480.0(↑)	710.6(↑)
Osteocalcin (ng/mL)	7.8–30.8	17.4	19.4(↑)	22.4(↑)
TRACP-5b (mU/dL)	120–420	414	487(↑)	572(↑)
Plasma pyrophosphate (nM)	1600–2500	–	–	2751
PLP (nmol/L)	20.5–151	–	–	20.2
PL (nmol/L)	8.8–53.7	–	–	63.1
PLP/PL	1–4.2	–	–	0.3
Urinary PEA (μmol/g Crea)	5.9–76.6	559.1	129.8(↓)	321.1(↓)
Motor function tests				
6MWT	–	632	620(↓)	–
Timed up and go test (*sec*)	–	4.9	5.1(↑)	–
Sit-to-stand test	–	13	28(↑)	–
Weighed arm lift test (R/L, kg)	–	31/32	40/45(↑)	–

SDS: Standard deviation score, SD: Standard deviation, BAP: Bone alkaline phosphatase, TRACP-5b: Tartrate-resistant acid phosphatase 5b, PLP: Pyridoxal 5′-phosphate, PL: Pyridoxal, PEA: Phosphoethanolamine, 6MWT: 6-min walking test, R: Right, L: Left.

Upward and downward arrows indicate increased and decreased values, respectively, relative to the values before asfotase alfa.

**Fig. 2:**
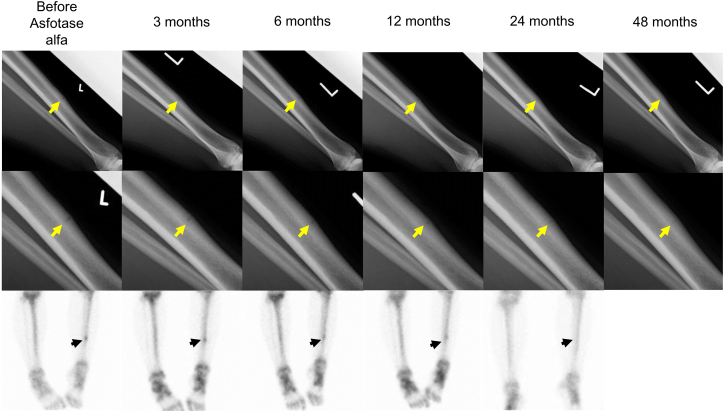
Radiographic improvement of a pseudofracture in the patient with HPP following asfotase alfa treatment (upper line: X-rays, middle line: enlarged images of X-rays, lower line: bone scintigraphy). Improvement of pseudofracture was detected by both X-ray (yellow arrows) and bone scintigraphy (black arrows). In the X-ray images, the radiolucent band became obscured but remained at one year and finally disappeared two years after the initiation of asfotase alfa. By bone scintigraphy, radionuclide accumulation began to diffuse at six months and disappeared as early as 24 months.

Radiological examinations performed before the initiation of asfotase alfa indicated a narrow radiolucent band and reactive thickening of the surrounding superficial bone in the middle diaphysis of her left tibia by X-ray and the accumulation of the nuclide at the same region by bone scintigraphy, which was compatible with the diagnosis of a pseudofracture (Fig. 2). No accumulation of nuclides was detected at anatomic sites other than the left tibia on bone scintigraphy. Because successful healing of the pseudofracture had not been achieved after a year of observation, asfotase alfa was initiated at a dose of 80 mg three times per week (6 mg/kg/week). The pain in the left tibia completely disappeared after two months of asfotase alfa treatment. Both X-ray and bone scintigraphy findings remained the same after three months. The accumulation of nuclides in the pseudofracture site with bone scintigraphy was significantly attenuated and decondensed after six months. Asfotase alfa was then reduced to 80 mg two times per week (4 mg/kg/week). The nuclide accumulation at the site completely disappeared after 24 months (Fig. 2). X-ray showed the complete union of the pseudofracture after 24 months, with a remaining responsive thickening of the bone surface (Fig. 2).

Physical function was assessed by a 6-min walk test (6MWT), timed up and go test (TUG), modified 30 s sit-to-stand test (30STS), and weighed arm lift test, as previously reported (Table 1) (Crapo et al., 2002; Podsiadlo and Richardson, 1991; Jones et al., 1999). The number of repetitions in 30STS doubled from 13 times at baseline to 28. The weighted arm lift test showed significant improvement on both sides from 31/32 (R/L, kg) to 40/45. Her baseline 6MWT and TUG were much higher than the average in the general population reported in a previous article (Genest et al., 2020), and there was no noticeable change after the treatment. For biochemical data parameters, the urinary PEA level decreased from 559.1 to 133.4 μmol/gCrea (reference range, 5.9–76.6) as early as one month after starting replacement therapy, and it continued to decrease to 321.1 μmol/gCrea. Plasma PP_i_, serum pyridoxal 5′-phosphate (PLP) and serum pyridoxal (PL) levels were measured after four years of treatment with asfotase alfa with a previously reported method (Akiyama et al., 2017; Jansen et al., 2013); PP_i_ was slightly higher than the upper value in the reference interval, and the PL level was elevated concomitant with decreased PLP levels and PLP/PL ratios, which suggested almost normalized enzymatic activity with asfotase alfa. The efficacy of asfotase alfa in pseudofracture healing and physical function improvement prompted us to continue replacement therapy.

## 3 Discussion

This report provides the detailed clinical course of radiological and physical improvements of a pseudofracture in an adult patient with benign prenatal HPP after initiating asfotase alfa. Before the initiation of replacement therapy with asfotase alfa, pseudofracture was detected by both X-ray and bone scintigraphy, accompanied by bone pain in the same region. Urinary PEA was significantly high, compatible with impaired activity in ALP. One month after replacement therapy, urinary PEA was significantly decreased but still slightly higher than the reference range. Three months after the treatment, while X-ray and bone scintigraphy still detected pseudofracture, local pain was alleviated. After six months of treatment, the nuclide accumulation began to diffuse. After one year, the nuclide accumulation was blurred but still recognizable, and union of the pseudofracture was not yet completed as shown by X-ray. On the other hand, several physical function tests, including the 30STS and weighed arm lift test, showed significant improvement at that time. Furthermore, X-ray revealed complete union of the pseudofracture, and the nuclide accumulation on bone scintigraphy disappeared after two years of treatment with asfotase alfa. Finally, after four years of treatment with asfotase alfa, the urinary PEA remained low but was still higher than the normal range, suggesting successful enzyme replacement. Although PPi, PLP, and PL levels before the treatment were not available, these parameters after four years of treatment also supported the strong efficacy of asfotase alfa.

In our report, the radiological alleviation of the pseudofracture was detected by X-ray and bone scintigraphy six months after the initiation of asfotase alfa, which was compatible with previous reports about adults with infantile- and adult-onset HPP (Klidaras et al., 2018; Magdaleno et al., 2019). Moreover, the patient experienced continuous improvement of the pseudofracture with no adverse events during the two years of asfotase alfa treatment, which was similar to the clinical course of adult-onset HPP in a previous case report (Klidaras et al., 2018). This study also tracked the patient's physical function using specific metrics and found significant improvements in the 30STS and weighed arm lift test after a year-long treatment with asfotase alfa. Finally, nearly normalized biochemical markers associated with HPP, such as serum plasma PP_i_, serum PLP, serum PL, and decreased urinary PEA, supported the efficacy of asfotase alfa in this patient.

To date, many reports have shown the remarkable efficacy of asfotase alfa in infants, children, adolescents, and adults with HPP (Kishnani et al., 2019; Whyte et al., 2016, Whyte et al., 2019). However, only a small number of studies have reported the clinical course of pseudofracture healing in response to asfotase alfa in adult patients with infantile-, pediatric- or adult-onset HPP. The pseudofracture typically developed in the immaturely mineralized bone tissue, and the detailed evaluation of the pseudofracture in pediatric-onset HPP under treatment with asfotase alfa, using three-dimensional cone-beam computed tomography, indicated the pathophysiologic changes of the remineralization in the osteoid tissue (Schilcher et al., 2019; Stürznickel et al., 2021). Rolvien et al. assessed the healing course of fracture in adults with infantile-onset HPP under treatment with asfotase alfa by several modalities, including physical function tests, magnetic resonance imaging (MRI), and dual-energy X-ray absorptiometry (Rolvien et al., 2019). They reported the rapid improvement of physical functions and bone edema with MRI 2 to 8 months after the initiation of asfotase alfa. Klidaras et al. reported the clinical courses of four fractures, including one pseudofracture, in two adults with infantile- and adult-onset HPP, and progressive healing of the pseudofracture was observed with X-ray at eleven months after the initiation of asfotase alfa (Klidaras et al., 2018). Magdaleno et al. reported a patient with adult-onset HPP whose bone scintigraphy revealed attenuation of uptake at the pseudofracture site after six months of treatment with asfotase alfa (Magdaleno et al., 2019). Kitaoka et al. reported the efficacy of asfotase alfa on fracture healing in a Japanese patient with adult-onset HPP harboring heterozygous c.1559delT, but the radiographic change of the fracture was not fully described (Kokaji et al., 2017). Compared to the abovementioned studies, this case report depicts both radiological and physical improvements following treatment with asfotase alfa and therefore could provide a better understanding of a pseudofracture healing process in patients with HPP.

There are some limitations to consider. First, several well-known biomarkers of HPP, such as PLP and PP_i_, were not available before asfotase alfa treatment, and we were not able to compare these biomarkers before and after enzymatic replacement therapy. Second, we evaluated only the 6MWT, TUG, 30STS and weighed arm lift test for the quantitative assessments of physical function; other guideline-recommended tools, including observational gait analysis, the Wong-Baker FACES Pain Rating Scale, EQ-5D-5L and SF-36, were not assessed in the current study (Kishnani et al., 2017). Third, although frequent visual evaluation by one-dimensional X-ray images and bone scintigraphy were performed on the pseudofracture site, there was no quantitative imaging evaluation conducted throughout the pseudofracture healing process in this case report. Fourth, the clinical course of our patient may not depict the typical pseudofracture healing process of patients with HPP treated with asfotase alfa, due to the reasons such as the differences in the affected region, the form of HPP, the age, sex and activity of the patients.

In conclusion, we report an adult patient with benign prenatal HPP whose pseudofracture was treated with asfotase alfa. We believe our detailed description of the radiological, physical and biochemical improvement after the initiation of asfotase alfa provides clinical insights to help physicians determine the optimal treatment of fractures/pseudofractures in HPP.

## Funding

This research did not receive any specific grant from funding agencies in the public, commercial, or not-for-profit sectors.

## Informed consent

Written informed consent was obtained from the patient, who has seen this report and has approved publication.

## CRediT authorship contribution statement

Hajime Kato: conceptualization, data curation, writing- original draft.

Naoko Hidaka: data curation, writing- review & editing.

Minae Koga: data curation, writing- review & editing.

Yuka Kinoshita: supervision, writing- review & editing.

Noriko Makita: supervision, writing- review & editing.

Masaomi Nangaku: supervision, writing- review & editing.

Nobuaki Ito: conceptualization, supervision, writing- review & editing.

## Declaration of competing interest

The authors declare that they have no known competing financial interests or personal relationships that could have appeared to influence the work reported in this paper.
